# Risk factors for progression of juvenile-onset non-radiographic axial spondyloarthritis to juvenile-onset ankylosing spondylitis: a nested case–control study

**DOI:** 10.1136/rmdopen-2021-001867

**Published:** 2021-12-03

**Authors:** Hao-Guang Li, Dan-Min Wang, Feng-Cai Shen, Shu-Xin Huang, Zhi-Duo Hou, Ling Lin, Zheng-Yu Xiao

**Affiliations:** 1 Department of Rheumatology and Immunology, The First Affiliated Hospital of Shantou University Medical College, Shantou, Guangdong, China; 2 Clinical Research Center, The First Affiliated Hospital of Shantou University Medical College, Shantou, Guangdong, China

**Keywords:** arthritis, juvenile, spondylitis, ankylosing, autoimmune diseases

## Abstract

**Objective:**

To evaluate the clinical characteristics of juvenile-onset non-radiographic axial spondyloarthritis (nr-axSpA) and to investigate risk factors associated with progression to juvenile-onset ankylosing spondylitis (JoAS).

**Methods:**

A nested case–control study was conducted using the retrospectively collected data of 106 patients with juvenile-onset nr-axSpA (age at disease onset, <16 years) in the Clinical characteristic and Outcome in Chinese Axial Spondyloarthritis study cohort. Baseline demographic and clinical characteristics and prognosis were reviewed. Logistic regression analyses were performed to investigate risk factors associated with progression to JoAS.

**Results:**

Overall, 58.5% of patients with juvenile-onset nr-axSpA presented with peripheral symptoms at disease onset. In 82.1% of these patients, axial with peripheral involvement occurred during the disease course. The rate of disease onset at >12 years and disease duration of ≤10 years were significantly higher in those with progression to JoAS than in those without progression to JoAS (83.0% vs 52.8%, p=0.001; 92.5% vs 56.6%, p<0.001, respectively). Multivariable logistic regression analysis revealed that inflammatory back pain (IBP) (OR 13.359 (95% CI 2.549 to 70.013)), buttock pain (OR 10.171 (95% CI 2.197 to 47.085)), enthesitis (OR 7.113 (95% CI 1.670 to 30.305)), elevated baseline C reactive protein (CRP) levels (OR 7.295 (95% CI 1.984 to 26.820)) and sacroiliac joint-MRI (SIJ-MRI) positivity (OR 53.821 (95% CI 9.705 to 298.475)) were significantly associated with progression to JoAS.

**Conclusion:**

Peripheral involvement was prevalent in juvenile-onset nr-axSpA. IBP, buttock pain, enthesitis, elevated baseline CRP levels and SIJ-MRI positivity in patients with the disease are associated with higher risk of progression to JoAS.

**Key messages**

**What is already known about this subject?**

- The clinical characteristics and long-term prognosis of adult-onset non-radiographic axial spondyloarthritis (nr-axSpA) have been previously elucidated. However, the clinical characteristics and disease course of juvenile-onset nr-axSpA are not well known.

**What does this study add?**

- Peripheral involvement was prevalent in juvenile-onset nr-axSpA; one should not expect typical axial skeleton involvement as the first clinical presentation in patients who are considered to have juvenile-onset nr-axSpA.
- Patients with juvenile-onset nr-axSpA with progression to juvenile-onset ankylosing spondylitis (JoAS) are more often >12 years of age at disease onset and have a disease duration of ≤10 years than those without progression to JoAS.
- Inflammatory back pain, buttock pain, enthesitis, elevated baseline C reactive protein levels and sacroiliac joint-MRI positivity in patients with juvenile-onset nr-axSpA are likely associated with stronger risk of progression to JoAS.

**How might this impact on clinical practice or further developments?**

- Our study findings enhance the current knowledge on juvenile-onset nr-axSpA. Future cohort studies including multiethnic and larger populations are warranted to delineate the long-term outcomes of juvenile-onset nr-axSpA.

## Introduction

Axial spondyloarthritis (axSpA) is a chronic inflammatory rheumatic disease that mainly affects the axial skeleton (sacroiliac joint and spine). It may involve the peripheral skeleton with extra-articular manifestations, including anterior uveitis, psoriasis and chronic inflammatory bowel disease. According to the 2009 Assessment of SpondyloArthritis International Society (ASAS) criteria,^1 2^ patients with axSpA are classified into the following two categories: those with radiographic axSpA (r-axSpA), known as ankylosing spondylitis (AS), who fulfil the modified New York criteria (mNYc)^3^ and those with non-radiographic axSpA (nr-axSpA) in the absence of definite sacroiliac joint (SIJ) changes on plain radiograph. However, it may take considerable time from the onset of clinical symptoms to fulfil the mNYc, which requires at least bilateral grade II or unilateral grade III radiographic changes in the SIJs. This contributes to diagnostic delay in AS.^4^ Therefore, the term nr-axSpA is proposed to enable earlier identification and appropriate clinical management of these patients.

Whether nr-axSpA represents an early stage of AS remains controversial.^5^ Clinical presentation, clinical disease activity (Bath Ankylosing Spondylitis Disease Activity Index) and treatment response are similar between the two subgroups of axSpA.^6 7^ This may support the hypothesis that nr-axSpA and AS are parts of the same disease. Several longitudinal cohort studies have shown that a proportion of cases with nr-axSpA progressed to AS, whereas others do not necessarily progress to AS during the follow-up period.^8–10^ This observation along with the identification of genetic and other differences between the two groups^11^—has led to the concept of nr-axSpA as a distinct disease entity.^12 13^ Multiple studies have investigated nr-axSpA prognosis to identify possible AS.^8–10^ These studies mainly focused on adult-onset nr-axSpA; therefore, the clinical characteristics, course and natural history of juvenile-onset nr-axSpA are not well known. Therefore, we conducted a nested case–control study to summarise the clinical characteristics of Chinese patients with juvenile-onset nr-axSpA and to investigate risk factors associated with progression to juvenile-onset ankylosing spondylitis (JoAS), with the overarching aim to further advance the current knowledge on juvenile-onset nr-axSpA.

## Patients and methods

Clinical characteristic and Outcome in Chinese Axial Spondyloarthritis (COCAS; registration no, ChiCTR2100049357) is a single-centre ambispective cohort study of patients with axSpA between the ages of 16 and 70 years. The COCAS study was designed to investigate factors related to radiographic progression and disease outcomes in Chinese patients with axSpA and was conducted at the Department of Rheumatology and Immunology and Clinical Research Center in the First Affiliated Hospital of Shantou University Medical College, China. The COCAS study included a retrospective phase (1999–2020) and a prospective (2021–2030) phase. The present nested case–control study used the retrospectively collected data between 1999 and 2019 from the COCAS cohort.

### Patients

A total of 199 patients with juvenile-onset nr-axSpA (age at disease onset,<16 years) were identified at baseline in the COCAS study cohort. Of these, 53 patients who progressed to JoAS with mNYc-positive radiographs after the enrollment comprised the case group. Controls were randomly selected from the remaining patients with nr-axSpA without mNYc-positive radiographs who were matched with cases at a ratio of 1:1 based on sex and enrollment time. All subjects had a follow-up duration of more than 1 year and had available a minimum of two pelvic radiographs obtained with more than 1year intervals.

### Study design

Clinical data recorded included gender, age at disease onset (age when the first disease-related symptom occurred), age at first visit, baseline disease duration (from onset of first symptoms to be classified as nr-axSpA), disease duration (from onset of first symptoms to the last follow-up visit), family history of SpA, clinical manifestations, human leucocyte antigen (HLA)-B27 status, erythrocyte sedimentation rate (ESR) (>15 and >20 mm/hour for males and females were defined as elevated), C reactive protein (CRP) level (>8 mg/L was defined as elevated), Ankylosing Spondylitis Disease Activity Score (ASDAS) (>2.1 was defined as high disease activity) and SIJ imaging assessment (on MRI and plain radiograph). A trained rheumatologist and radiologist independently assessed the images. In case of disagreement, a decision on the presence or absence of findings was achieved by another experienced rheumatologist. Definite radiographic sacroiliitis (grade II bilaterally or grade III–IV unilaterally) according to the mNYc 1984.^3^ SIJ-MRI was considered positive if bone marrow oedema (BME) lesions highly suggestive of SpA were present (either one BME lesion on ≥2 consecutive slides or several BME lesions on one slide).^14^ Enthesitis was defined as tenderness at the insertion point of a ligament or tendon to bone on palpation.^15^ The ASAS criteria were used to define inflammatory back pain (IBP).^16^

### Statistical methods

SPSS for Windows, V.26.0 (SPSS Inc IMB Company) was used for data analysis. Kolmogorov-Smirnov test of normality was performed for continuous data. Variables were presented as means with SD and medians with IQR for normally and non-normally distributed variables, respectively. Frequency (%) was given for counts. Mann-Whitney test was used to compare continuous values between groups. χ^2^ test or Fisher’s exact test was used to compare categorical variables, such as proportions, between groups. Univariable and multivariable logistic regression analyses were used to investigate risk factors associated with progression to JoAS. ORs with 95% CIs were calculated. Variables identified in the univariate analysis (p<0.10) were entered into a forward stepwise multiple logistic regression model. P values of <0.05 were considered statistically significant.

## Results

### Case–control study

Table 1 shows the baseline demographic and clinical characteristics of 106 patients with juvenile-onset axSpA. The patients with progression to JoAS had a shorter median baseline disease duration and significantly older median age at disease onset (3.0 (IQR 2.7) vs 5.0 (IQR 5.5), p<0.001; 14.0 (IQR 2.0) vs 13.0 (IQR 3.0), p=0.001, respectively) than those without progression to JoAS. There were significantly higher proportions of patients aged >12 years at disease onset, and a disease duration of ≤10 years was observed in patients with progression to JoAS than in those with nr-axSpA (83.0% vs 52.8%, p=0.001; 92.5% vs 56.6%, p<0.001, respectively). No significant difference was observed between the two groups in terms of the prevalence of HLA-B27 positivity and family history of SpA positivity (p>0.05).

**Table 1:** Baseline demographics of 106 cases of juvenile-onset axial spondyloarthritis

Characteristics*		Total (n=106)	nr-axSpA group (n=53)	JoAS group (n=53)	P value†
Male, n (%)		75 (70.8)	37 (69.8)	38 (71.7)	0.831
Age at first visit, (years)		17.0 (2.0)	17.0 (3.0)	16.0 (1.0)	0.024‡
Age at disease onset, (years)		14.0 (3.0)	13.0 (3.0)	14.0 (2.0)	0.001‡
Age at disease onset >12 years, n (%)		72 (67.9)	28 (52.8)	44 (83.0)	0.001
Baseline disease duration (years)		4.0 (4.0)	5.0 (5.5)	3.0 (2.7)	<0.001‡
Disease duration (≤10 years)§, n (%)		79 (74.5)	30 (56.6)	49 (92.5)	<0.001
HLA-B27 positive, n (%)		78 (73.6)	43 (81.1)	35 (66.0)	0.078
Family history of SpA, n (%)		26 (24.5)	13 (24.5)	13 (24.5)	1.000
Initial symptoms, n (%)	Axial	42 (39.6)	24 (45.3)	18 (34.0)	0.233
	Peripheral	62 (58.5)	27 (50.9)	35 (66.0)	0.115
	Extra-articular	2 (1.9)	2 (3.8)	0	0.475
Symptom, ever, n (%)	Axial only	19 (17.9)	13 (24.5)	6 (11.3)	0.076
	Axial and peripheral	87 (82.1)	40 (75.5)	47 (88.7)	0.076
	Extra-articular	11 (10.4)	6 (11.3)	5 (9.4)	0.750
Elevated ESR (baseline), n (%)		46 (43.4)	14 (26.4)	32 (60.4)	<0.001
Elevated CRP (baseline), n (%)		50 (47.2)	14 (26.4)	36 (67.9)	<0.001
ASDAS-ESR >2.1, n (%)¶		57 (75.0)	21 (60.0)	36 (87.8)	0.005
ASDAS-CRP >2.1, n (%)¶		57 (75.0)	19 (54.3)	38 (92.7)	<0.001
Clinical arm, n (%)		52 (49.1)	41 (77.4)	11 (20.8)	<0.001
Imaging arm, n (%)		54 (50.9)	12 (22.6)	42 (79.2)	
SIJ-MRI positivity, n (%)**		54 (56.3)	12 (26.1)	42 (84.0)	<0.001

*Values are median (IQR), unless otherwise indicated.

†χ^2^ test was used to compare the nr-axSpA and JoAS group, unless otherwise indicated.

‡Mann-Whitney U test.

§Disease duration: from onset of symptoms to the last follow-up visit.

¶Available in 76 patients (n=35 for the nr-axSpA group and n=41 for the JoAS group).

**Available in 96 patients (n=46 for the nr-axSpA group and n=50 for the JoAS group).

ASDAS-CRP, Ankylosing Spondylitis Disease Activity Score (C reactive protein); ASDAS-ESR, Ankylosing Spondylitis Disease Activity Score (erythrocyte sedimentation rate); CRP, C reactive protein; ESR, erythrocyte sedimentation rate; HLA-B27, human leucocyte antigen B27; IBP, inflammatory back pain; JoAS, juvenile-onset ankylosing spondylitis; NI, not included in the multivariate model; nr-axSpA, non-radiographic axial spondyloarthritis; SIJ-MRI, sacroiliac joint-MRI; SpA, spondyloarthritis.

More than half (58.5%) of the patients with juvenile-onset nr-axSpA presented with peripheral symptoms at disease onset, but extra-articular manifestations were observed only in 1.9%. Axial involvement with peripheral involvement occurred in a great percentage of these patients (82.1%) during the disease course. Axial involvement alone accounted for 17.9%, whereas extra-articular manifestations were present in 10.4% patients.

The proportions of patients with elevated CRP level and ESR were significantly higher among patients with progression to JoAS than in those with nr-axSpA (67.9% vs 26.4%, p<0.001; 60.4% vs 26.4%, p<0.001, respectively), as were the ASDAS-CRP >2.1 and ASDAS-ESR >2.1 (92.7% vs 54.3%, p<0.001; 87.8% vs 60.0%, p=0.005, respectively). There were 54 (50.9%) patients who showed active sacroiliitis with BME lesions on MRI. They were classified as nr-axSpA using the ‘imaging arm’ of the ASAS criteria. The remaining 42 (39.6%) who did not show active sacroiliitis on MRI and the other 10 (9.4%) whose MRIs were unavailable could only be classified as having nr-axSpA using the ‘clinical arm’ of the ASAS classification criteria. The percentage of SIJ-MRI positivity was significantly higher in patients with progression to JoAS than in those with nr-axSpA (84.0% vs 26.1%, p<0.001).

**Figure 1:**
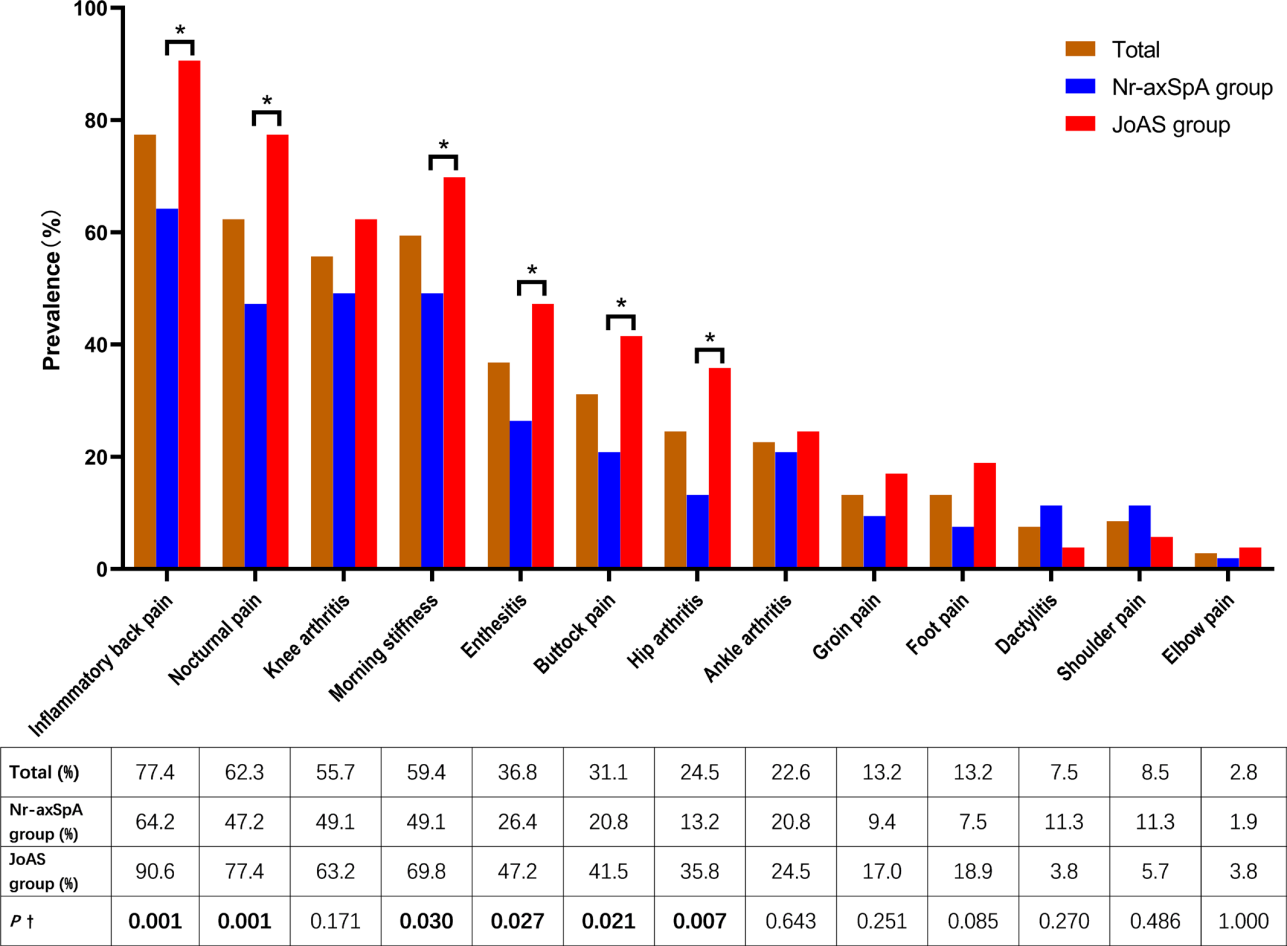
Clinical manifestations of juvenile-onset axial spondyloarthritis (axSpA). ^†^χ^2^ test was used to compare the juvenile-onset non-radiographic axial spondyloarthritis (nr-axSpA) and juvenile-onset ankylosing spondylitis (JoAS) groups. *There was a statistically significant difference between the nr-axSpA and JoAS groups (p<0.05). Inflammatory back pain, buttock pain, hip arthritis, enthesitis, morning stiffness and nocturnal pain occurred more often in patients with progression to JoAS than in nr-axSpA (90.6% vs 64.2%, p=0.001; 41.5% vs 20.8%, p=0.021; 35.8% vs 13.2%, p=0.007; 47.2% vs 26.4%, p=0.027; 69.8% vs 49.1%, p=0.030; 77.4% vs 47.2%, p=0.007, respectively).

Figure 1 shows the clinical manifestations of 106 patients with juvenile-onset axSpA. The most common clinical manifestation was IBP (77.4%) followed by knee arthritis (55.7%) and then enthesitis (36.8%). The presence of IBP, buttock pain, hip arthritis, enthesitis, morning stiffness and nocturnal pain was more frequent in patients with progression to JoAS than in those with nr-axSpA (90.6% vs 64.2%, p=0.001; 41.5% vs 20.8%, p=0.021; 35.8% vs 13.2%, p=0.007; 47.2% vs 26.4%, p=0.027; 69.8% vs 49.1%, p=0.030; 77.4% vs 47.2%, p=0.007, respectively).

### Risk factors of progression to JoAS

During the follow-up of 12 (median 3.0 (IQR 3.0)) years, 53 patients progressed to JoAS in the COCAS cohort. In the univariable logistic regression analysis to investigate risk factors associated with progression to JoAS, the variables of age at disease onset, disease duration, morning stiffness, nocturnal pain, buttock pain, IBP, hip arthritis, enthesitis, baseline elevated acute-phase reactants (CRP and ESR) and MRI-positive sacroiliitis were statistically significant (p<0.05). Variables identified in the univariate analysis were subsequently entered into a forward stepwise multiple logistic regression model. The final multivariable model analysis revealed that IBP, buttock pain, enthesitis, elevated baseline CRP levels and SIJ-MRI positivity were statistically significant associations with progression to JoAS (table 2).

**Table 2:** Risk Factors for the progression of juvenile-onset nr-axSpA to JoAS in univariable and multivariable analyses*

Risk factor	Univariable analysis		Multivariable analysis model	
	OR (95% CI)	P value	OR (95% CI)	P value
Male	1.095 (0.474 to 2.531)	0.831	NI	
Disease onset (age >12 years)	4.365 (1.780 to 10.706)	0.001	NI	
Disease duration (≤10 years)†				
≤5 years	10.925 (2.950 to 40.453)	<0.001	NI	
<5 and ≤10 years	8.625 (2.590 to 28.726)	<0.001	NI	
>10 years	1.0 (reference)	–		
Family history of SpA	1.000 (0.413 to 2.423)	1.000	NI	
HLA-B27 positivity	0.452 (0.185 to 1.104)	0.081	NI	
Morning stiffness	2.401 (1.083 to 5.324)	0.031	NI	
Nocturnal pain	3.827 (1.653 to 8.859)	0.002	NI	
Axial symptoms, ever				
Buttock pain	2.710 (1.147 to 6.402)	0.023	10.171 (2.197 to 47.085)	0.003
IBP	5.365 (1.824 to 15.776)	0.002	13.359 (2.549 to 70.013)	0.002
Peripheral arthritis, ever				
Knee	1.713 (0.790 to 3.714)	0.172	NI	
Ankle	1.241 (0.498 to 3.090)	0.643	NI	
Hip	3.672 (1.387 to 9.720)	0.009	NI	
Enthesitis, ever	2.487 (1.101 to 5.617)	0.028	7.113 (1.670 to 30.305)	0.008
Elevated CRP (baseline)	5.899 (2.547 to 13.664)	<0.001	7.295 (1.984 to 26.820)	0.003
Elevated ESR (baseline)	4.245 (1.866 to 9.658)	0.001	NI	
SIJ-MRI positivity	13.045 (5.176 to 32.879)	<0.001	53.821 (9.705 to 298.475)	<0.001

*All variables with a p value below 0.10 from the univariable analysis were entered into a multivariable analysis model (forward logistic regression model).

†Disease duration: from onset of symptoms to the last follow-up visit.

CRP, C reactive protein; ESR, erythrocyte sedimentation rate; HLA-B27, human leucocyte antigen B27; IBP, inflammatory back pain; JoAS, juvenile-onset axial spondyloarthritis; NI, not included in the multivariate model; nr-axSpA, non-radiographic axial spondyloarthritis; SIJ-MRI, sacroiliac joint-MRI; SpA, spondyloarthritis.

## Discussion

To our knowledge, this is the first study to investigate the demographics, clinical features and risk factors associated with progression to JoAS in Chinese patients with juvenile-onset nr-axSpA.

Patients with nr-axSpA enrolled in our study were 16 years or older at their first visit. According to the Chinese regulations, these patients should visit the adult Department of Rheumatology but not the paediatric department. Therefore, the ASAS classification criteria were used to classify these cases as having nr-axSpA. However, because these patients developed the disease before the age of 16 years, their clinical features and outcomes may be different from those of adult-onset nr-axSpA. Analysis of the clinical features and prognosis of juvenile-onset nr-axSpA may advance current understanding of the disease.

Our data showed that juvenile-onset nr-axSpA is more common in males (male:female=2.4:1), whereas adult-onset nr-axSpA usually exhibits a comparable sex ratio. Axial spondyloarthritis is strongly associated with HLA-B27 positivity, and the HLA-B27 was positive in 73.6% patients in our study, which is consistent with a previous study that reported HLA-B27 prevalence ranging from 37% to 90%.^5^ Patients with progression to JoAS had shorter duration of baseline disease because they were older at disease onset and younger at diagnosis than those without progression to JoAS. Furthermore, in patients aged >12 years at disease onset the proportion of those who progressed to JoAS was 61.1%, but this decreased to 26.5% when the age at onset was ≤12 years (p=0.001), indicating that age at disease onset may be associated with progression to JoAS. These data are consistent with earlier studies that reported that the average age at the onset of JoAS was higher than 12 years.^17–19^ Interestingly, the proportion of those who progressed from nr-axSpA to JoAS was approximately four times higher in patients with a shorter duration of disease (≤10 years) than in patients with a longer duration of disease (>10 years). These data suggest that patients with juvenile-onset nr-axSpA and a long disease duration of >10 years who still do not exhibit radiographic structural SIJ damage, the presence of which would fulfil the mNYc, are less likely to progress to JoAS in the future; however, the association between the disease duration and radiographic progression remains to be explored.

Although the axial skeleton may be involved in the disease course, peripheral arthritis was the most common first symptom of juvenile-onset nr-axSpA.^20^ This suggests that the disease onset pattern of juvenile-onset nr-axSpA presents as a ‘peripheral predominant’ mode, consistent with earlier cross-sectional studies that compared JoAS and adult-onset AS.^18^ Goirand *et al* analysed 114 French patients with peripheral spondyloarthritis/enthesitis-related arthritis in a median 2.5-year follow-up. They found that axial disease and sacroiliitis were rare at disease onset, but appeared during follow-up in 63% and 47% of cases, respectively.^21^ Therefore, one should not expect typical axial skeleton involvement as the first clinical presentation when patients are considered as having juvenile-onset nr-axSpA.

ASDAS, which includes objective inflammatory markers (serum ESR/CRP concentration) and subjective assessments of disease activity, was used as clinical tools for measuring disease activity in patients with juvenile-onset nr-axSpA. The well performance of ASDAS has been demonstrated in the assessment of the condition of patients with adult-onset SpA.^22^ However, because ASDAS is predominantly focused on axial symptoms and showed a lower weight to the evaluation of peripheral symptoms, it may not perform well in assessing the disease activity of patients with juvenile-onset nr-axSpA because the peripheral involvement occurred in a great percentage of these patients (82.1%) during the disease course in this study. Moreover, it does not specifically include the measure of enthesitis. Follow-up validation might require inclusion of other items as identified by key stakeholders, as well as longitudinal evaluations to improve the precision and responsiveness of the tool.

Analysis of the German Spondyloarthritis Inception Cohort (GESPIC) found that of 95 patients with nr-axSpA, 11.6% progressed to AS over 2 years.^9^ Another cohort study by our team showed that over the period from 5 to 10 years, 18 (52.9%) of 34 patients with pathological evidence of sacroiliitis progressed to AS; meanwhile, there was no progression to AS in patients with negative pathological sacroiliitis over 0.3–8.5 years.^8^ A recent population-based cohort study from the USA reported that 16 (26.4%) of 83 subjects had nr-axSpA progression to AS over a mean follow-up of 10.6 years.^10^ These findings revealed that not all patients with nr-axSpA will progress to AS. Therefore, identifying ‘early AS’ is of great significance in clinical practice. During the follow-up of 12 years in our study, there was a significantly higher percentage of SIJ-MRI positivity among patients with progression to JoAS than in patients with nr-axSpA, this result supports the conclusion reported by previous studies regarding MRI evidence of sacroiliitis as a marker for progression of nr-axSpA to AS.^23^ Therefore, active sacroiliitis on MRI (SIJ-MRI positivity) could predict the progression of juvenile-onset nr-axSpA to JoAS. In the absence of imaging evidence suggesting sacroiliitis, caution is warranted when considering a case as ‘early AS’.

Besides the presence of IBP, buttock pain and enthesitis were associated with progression to JoAS in the multivariate analysis in our study. Our data also showed that elevated CRP level was associated with progression to JoAS, but not ESR. This is consistent with the GESPIC study that reported that an elevated CRP level at baseline was a strong positive predictor of radiographic sacroiliitis progression in adult-onset nr-axSpA.^9^

Classification of juvenile-onset axSpA remains challenging unlike adult-onset cases. In paediatric rheumatology, these patients may be categorised into the subtype of juvenile idiopathic arthritis as defined by the International League of Associations for Rheumatology classification criteria^24^; however, these classification systems do not include sensitive imaging tools—such as MRI—which has been confirmed to be able to predict the progression of juvenile-onset nr-axSpA to JoAS in our research. However, clinical application of SIJ-MRI in children is still challenging. It is critical to understand the normal features of the immature SIJ-MRI before inflammatory changes can be accurately identified. Further studies are needed to determine the role of MRI in the classification criteria for juvenile-onset SpA, as well as to refine pediatric-specific definitions of a ‘positive’ MRI in juvenile-onset SpA.

This study has several limitations. First, the sample size is small. Second, this was a single-centre, retrospective study, which introduces several important limitations. For example, some patients lacked information on ASDAS or SIJ-MRI at baseline. Third, the follow-up duration was heterogeneous, ranging from 1 to 12 years and the long-term outcomes of patients with nr-axSpA who were followed for shorter than 2 years do not allow definite conclusions.

## Conclusion

In summary, peripheral involvement was prevalent in juvenile-onset nr-axSpA; therefore, one should not expect typical axial skeleton involvement as the first clinical presentation when patients are considered to have juvenile-onset nr-axSpA. Patients with progression to JoAS more often are >12 years at disease onset and have a disease duration of ≤10 years than those without progression to JoAS. IBP, buttock pain, enthesitis, elevated baseline CRP levels and SIJ-MRI positivity in patients with juvenile-onset nr-axSpA are likely to be associated with higher risks of progression to JoAS.

## Data Availability

Data are available upon reasonable request.
